# Yersinia pseudotuberculosis infection in Kawasaki disease and its clinical characteristics

**DOI:** 10.1186/s12887-015-0497-2

**Published:** 2015-11-11

**Authors:** Tomoko Horinouchi, Kandai Nozu, Kiyoshi Hamahira, Yosuke Inaguma, Jun Abe, Hiroshi Nakajima, Masaaki Kugo, Kazumoto Iijima

**Affiliations:** Department of Pediatrics, Japanese Red Cross Society Himeji Hospital, Hyogo, Japan; Department of Pediatrics, Kobe University Hospital, Kobe, Japan; Department of Allergy and Immunology, National Research Institute for Child Health and Development, Tokyo, Japan; Department of Bacteriology, Okayama Prefectural Institute for Environmental Science and Public Health, Okayama, Japan

## Abstract

**Background:**

The etiology of Kawasaki disease (KD) is unknown. Reportedly, there is an association between KD and *Yersinia pseudotuberculosis* (YPT). Steroid therapy for KD patients with high risk of cardiac sequelae (CS) has been reported; however, the number of reports is limited.

**Methods:**

We conducted a prospective study of 108 patients with newly diagnosed KD in one year to determine how many KD patients have positive anti-YPT antibody titers and/or positive anti-YPT-derived mitogen (YPM) antibody titers. In addition, we tried to identify clinical differences between KD patients in whom YPT infection was or not a contributing factor. We also compared clinical characteristics of patients treated with the protocol of the Randomized controlled trial to Assess Immunoglobulin plus Steroid Efficacy for Kawasaki disease (RAISE) study (RAISE group) and with the conventional Intravenous immunoglobulin (IVIG) protocol (conventional group).

**Results:**

Eleven patients (10 %) were positive for anti-YPT and/or anti-YPM antibodies (positive group) and 97 (90 %) were negative (negative group). Cardiac sequelae (CS) occurred significantly more frequently in the positive than the negative group (two patients, 18 % vs one patient, 1 %, *p* = 0.027). Forty patients were in the RAISE group. Two of 40 (5 %) in the RAISE group and one of 68 (1.47 %) in the conventional group had CS (*p* = 0.55).

**Conclusions:**

KD patients with YPT infection had CS significantly more frequently and treatment with RAISE protocol did not decrease the frequency of CS in our cohort, nor did YPT infection affect risk scores of no response to IVIG. However, our sample size was overly small to draw such conclusions. Further investigation in a larger cohort is necessary to confirm our findings. Additionally, further research is needed to determine whether early diagnosis of YPT can prevent KD from developing and reduce the incidence of CS.

## Background

Kawasaki disease (KD), a febrile vasculitis of unknown origin, can cause coronary artery dilation and is increasing in incidence worldwide [1–3]. Identification of the cause and optimal treatment is urgently needed.

*Yersinia pseudotuberculosis* (YPT), an enteric pathogen, causes a variety of clinical symptoms such as fever, rash, desquamation, strawberry tongue, lymphadenopathy, and conjunctivitis that sometimes satisfy the clinical criteria for KD. Some research groups have reported an association between YPT and KD [4–7]. YPT is commonly typed serologically according to antigenic differences in the lipopolysaccharide O antigen. There are 15 serogroups; serotypes O:1 and O:2 have subtypes a, b, and c, and serotypes O:4 and O:5 have subtypes a and b. Thus, there are 21 serotypes in all [8, 9]. Serotypes O:1a, O:1b, O:2a, O:2b, O:2c, O:3, O:4b, O:5a, O:5b, O:6, O:10, and O:15 are known to be pathogenic for humans [10]. The pathogenicity of YPT is determined by a number of virulence factors, including a plasmid associated with *Yersinia* virulence, a high-pathogenicity island, and a *Y. pseudotuberculosis*-derived mitogen (YPM) [11–13]. Among these virulence factors, YPM is a superantigen that activates macrophages and T cells and causes symptoms such as high fever and erythema [14].

So far, few reports concerning the relationship between KD and YPT infection are available. The present study was designed to determine how often KD patients have associated YPT infection by assessing anti-YPT and anti-YPM antibody titers and to identify clinical differences between KD patients with and without YPT infection.

Additionally, about 40 % of KD patients in our cohort were treated with the RAISE (Randomized controlled trial to Assess Immunoglobulin plus Steroid Efficacy for Kawasaki disease) study protocol [15]. Clinical characteristics of patients treated with the RAISE protocol and the conventional IVIG (IntraVenous ImmunoGlobulin) protocol were compared to assess the effects of the RAISE study treatment strategy in patients with KD and the influence of YPT infection on treatment outcomes.

## Methods

### Study patient definitions and design

With approval of the Japanese Red Cross Society Himeji Hospital ethics committee, all KD patients hospitalized from April 2013 to March 2014 in the Japanese Red Cross Society Himeji Hospital were prospectively included in this study. Written informed consent for not only participating in this study but also to publish the patient data and information was obtained from all parents or guardians. Patients whose guardians refused consent or for whom blood samples for detection of anti-YPT and YPM antibodies were not available were excluded. Acute- and convalescent-phase sera were obtained from participants, who were then divided into two groups: a positive group (positive anti-YPT and/or positive anti-YPM antibody titers) and a negative group (both anti-YPT and anti-YPM antibody titers negative).

The following clinical data were recorded and compared between the two groups: sex, age, duration of fever in days, frequency of administration of high-dose IVIG, acute-phase coronary artery dilation, cardiac sequelae (CS), abdominal symptoms, white blood cell and absolute neutrophil counts, serum concentrations of albumin, total bilirubin, aspartate aminotransferase, sodium, C-reactive protein (CRP), procalcitonin, N-terminal pro-brain natriuretic peptide, and soluble interleukin-2 receptor, and urinary β2-microglobulin/creatinine ratio.

### Measurement of anti-YPT antibody and anti-YPM antibody

To identify and quantitate anti-YPT antibodies, lipopolysaccharide agglutination titers for serotypes O:1a, 1b, 2a, 2b, 3, 4a, 4b, 5a, 5b, and 6 were measured by the Widal method. Sera positive for O:2 and/or O:4 were retested after absorption with *Salmonella* O groups four and nine, respectively, because the former have cross-O-antigen reaction against the latter [16, 17]. The cut-off for positive anti-YPT antibodies was a single titer of 1:160 or higher [10].

Enzyme-linked immunosorbent assays were performed to measure anti-YPM antibodies, the optical density of non-antigen-coated wells being subtracted from that of antigen-coated wells. Anti-YPM antibody titers were determined using a calibration curve constructed from a positive control serum. Patients with a more than four-fold increase in serial antibody titers were considered positive for anti-YPM antibodies.

### Definition and treatments for KD

**Fig. 1:**
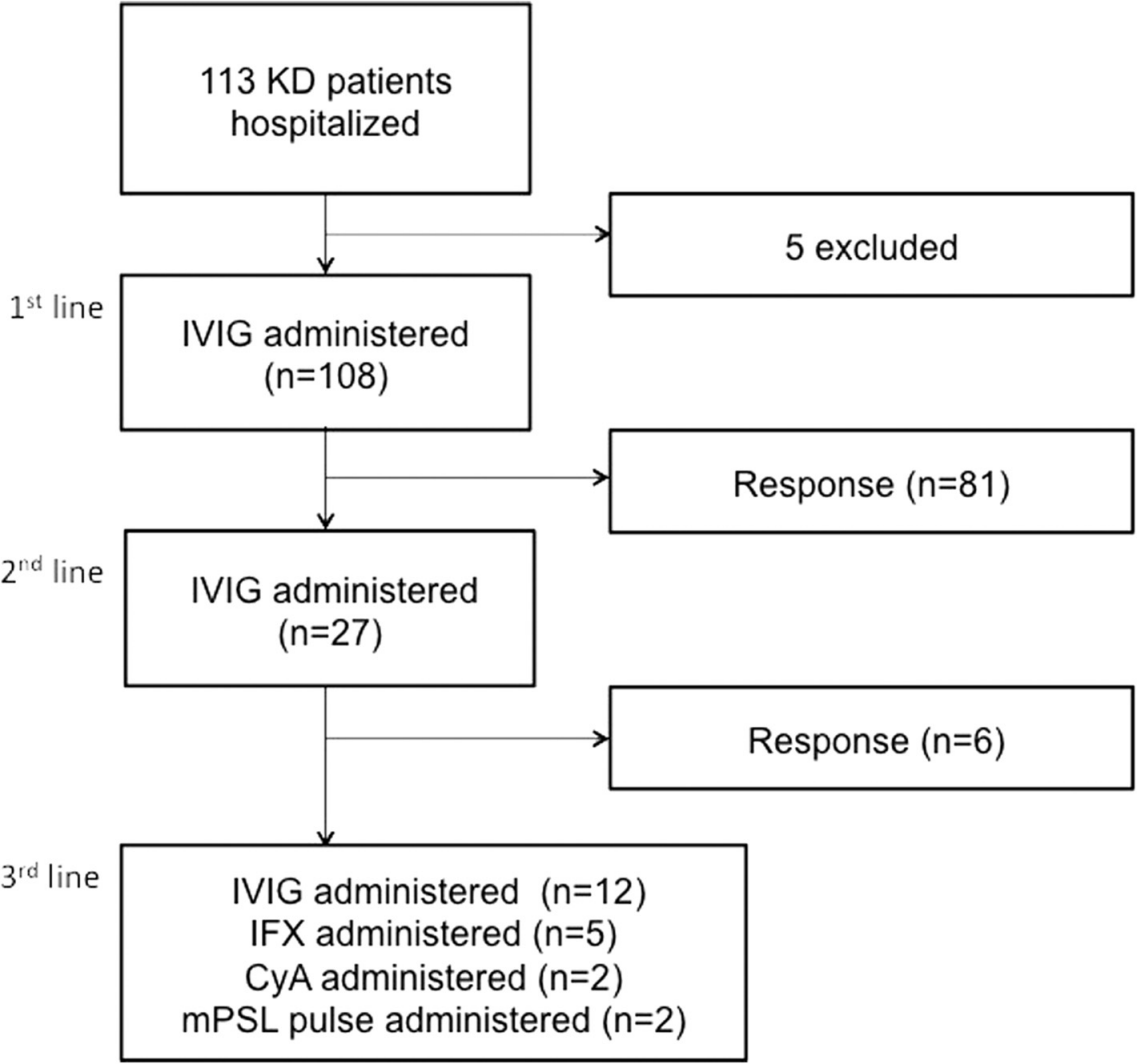
Flow chart showing distribution of study subjects and medications used for KD. The numbers in parentheses denote the overall number of patients. KD: Kawasaki disease, IVIG: IntraVenous ImmunoGlobulin, IFX: Infliximab, CyA: cyclosporin, mPSL: methylprednisolone

Kawasaki disease was diagnosed in accordance with the Japanese Diagnostic Guidelines for Kawasaki Disease (5th edition) [17]. The initial treatment for KD consisted of IVIG (2 g/kg) (*n* = 108). A second dose of IVIG (2 g/kg) was given to patients whose fever had not resolved ≥48 h after receiving the first IVIG (*n* = 27). For patients whose fever had not resolved ≥48 h after receiving a second IVIG, additional treatments, including a third administration of IVIG (2 g/kg) (*n* = 12), infliximab (5 mg/kg) (*n* = 5), cyclosporin (4 mg/kg per day, until CRP concentrations had reverted to normal) (*n* = 2), or methylprednisolone (30 mg/kg per day, for 3 days) (*n* = 2) were considered. No patient underwent plasma exchange (Fig. 1). Almost all patients received aspirin 30 mg/kg per day until they were afebrile, followed by aspirin 3 mg/kg per day for 2 months after discharge from the hospital. Flurbiprofen (4 mg/kg/day) was substituted in patients with serum aspartate aminotransferase or alanine aminotransferase concentrations ≥200 U/L.

Coronary artery dilation was defined as the internal diameter of the coronary artery being >3 mm (>4 mm if the subject was over 5 years old) or the internal diameter of a segment being at least 1.5 times that of an adjacent segment according to echocardiography [18]. Acute-phase coronary artery dilations were defined as those that normalized within 1 month and CS were defined as those remaining after 1 month [15].

### RAISE study group

The treatment strategy was changed during the study period. At first, we started the KD treatment with the conventional IVIG protocol. However, we decided to change the treatment protocol, that is, we adopted the RAISE protocol, which reported clear evidence that steroids prevent CS in KD patients [15] and has already been adopted as a standard treatment in Japan which reflects the fact that this protocol has recently described in the guideline for treatment of KD in Japan [18, 19].

In response to the findings of the RAISE study, between December 2013 and March 2014, patients with risk scores ≥5 points, which predicts failure to respond to initial treatment with IVIG, we adopted the RAISE protocol which reported clear evidence that intravenous prednisolone (PSL) 2 mg/kg per day (maximum dose of 60 mg per day) prevents CS in KD [15]. Once patients had become afebrile, PSL was administered orally. When serum CRP concentrations had decreased to within the normal range (≤0.5 mg/dL), the PSL dose was tapered over 15 days with the following dosage reductions at 5 day intervals: from 2 mg/kg per day to 1 mg/kg per day to 0.5 mg/kg per day. Scoring and cut-off values for risk scores were measured as described in the RAISE study, which is as follows: two points each for serum sodium concentration of ≤133 mmol/L, ≤4 days of illness at diagnosis, serum aspartate aminotransferase concentration of ≥100 U/L, neutrophil count of ≥80 %; and 1 point each for platelet count ≤30 × 10^4^/μL, serum CRP concentration of ≥10 mg/dL, and age ≤12 months [15]. Patients with risk scores of ≥5 points were regarded as high risk. Patients who were treated according to the RAISE study protocol were defined as the RAISE group and the others were defined as the conventional group.

### Statistical analysis

The Mann–Whitney *U* test was performed to compare continuous variables between these two groups and Fisher’s exact test for categorical variables. Differences were considered significant if *p* < 0.05. Data are expressed as median and interquartile range. All calculations were performed with standard statistical software (JMP version 11 package for Windows, SAS, Cary, NC, USA).

## Results

During the year of the study, 113 patients with KD were admitted to our hospital. After excluding five patients, one because their guardian refused consent and four for whom blood samples were not available, 108 patients were included in the study (Fig. 1).

Eleven patients (10 %) were in the positive group, and 97 (90 %) in the negative group. Of the eleven patients in the positive group, eight were positive only for anti-YPT antibodies, one for only anti-YPM antibody and two for both (Table 1). Patients 7 (O:2b, 5b), 9 (O:5a, 5b), and 11 (O:1b, 2b) had increases in titers of more than two types of antibody.

**Table 1:** Characteristics of patients in positive group

No	Age (months)	Sex	YPT	YPM	CS	Treatment group	Risk group
1	19	Male	O6 (1:640)	Negative	(−)	Conventional	Low
2	29	Female	O6 (1:160)	Negative	(−)	Conventional	Low
3	72	Male	O3 (1:640)	Negative	(−)	Conventional	Low
4	6	Male	Negative	Positive	(−)	Conventional	Low
5	66	Male	O5b (1:160)	Negative	(+)	Conventional	High
6	34	Female	O6 (1:160)	Negative	(−)	RAISE	Low
7	22	Male	O2b,5b (1:160)	Negative	(+)	RAISE	High
8	70	Male	O1a (1:160)	Negative	(−)	RAISE	Low
9	21	Female	O5a,5b (1:160)	Positive	(−)	RAISE	Low
10	79	Female	O6 (1:160)	Negative	(−)	RAISE	High
11	21	Female	O1b,2b (1:160)	Positive	(−)	RAISE	Low

Clinical manifestations of patients with KD according to serologically positive and negative groups are compared in Table 2. The only significant differences between the two groups were in the incidence of CS (positive group, two patients [18 %] vs negative group, one patient [1 %]; *p* = 0.027) and of abdominal symptoms (positive group, eight patients [73 %] vs negative group, 33 patients [34 %]; *p* = 0.020).

**Table 2:** Comparison of characteristics according to serologically positive and negative groups

	Positive group (*n* = 11)	Negative group (*n* = 97)	*P* value
Sex (male/female)	6/5	61/36	0.74
Age (months)	29 (21–70)	24 (13–49.5)	0.26
Duration of fever (days)	6 (6–9)	5 (5–7)	0.092
Frequency of administration of IVIG	1 (1–2)	1 (1–1)	0.32
Acute-phase coronary artery dilation; n (%)	2 (18.18 %)	4 (4.12 %)	0.11
CS; n (%)	2 (18.18 %)	1 (1.03 %)	0.027
Abdominal symptoms; n (%)	8 (72.73 %)	33 (34.02 %)	0.02
White blood cell count (/*μ* L)	12,900 (11,100–19,400)	13,000 (10,750–15,400)	0.48
Absolute neutrophil count (/*μ* L)	8117 (5741–24,654)	8032 (5696–10,240)	0.73
Serum albumin concentration (g/dL)	3.8 (3.4–4)	3.7 (3.4–4)	0.54
Serum total bilirubin concentration (mg/dL)	0.4 (0.3–0.7)	0.6 (0.4–0.8)	0.47
Serum aspartate aminotransferase (U/L)	37 (19–230)	32.5 (25–69.25)	0.8
Serum sodium (mmol/L)	134 (133–138)	136 (134–138)	0.24
Serum CRP concentration (mg/dL)	4.36 (1.81–8.09)	7.09 (3.09–11.72)	0.27
Serum procalcitonin concentration (ng/mL)	0.67 (0.42–2.18)	0.47 (0.175–1.995)	0.37
N-terminal pro-brain natriuretic peptide (pg/mL)	379 (81.75–771.5)	386 (140–936)	0.74
Serum soluble interleukin-2 receptor (U/mL)	1560 (1350–2680)	1635 (1062.5–2157.5)	0.37
Urinary *β* 2-microglobulin/creatinine ratio	101.9 (10.7–203.7)	18.9 (5.9–126.3)	0.25
High risk patients; n (%)	3 (27.27 %)	26 (27.08 %)	1

Values expressed as count (%) for categorical variables and median (IQR) for continuous variables

Although blood and/or stool cultures were performed in many patients in this study, YPT was not cultured from any of the samples.

**Fig. 2:**
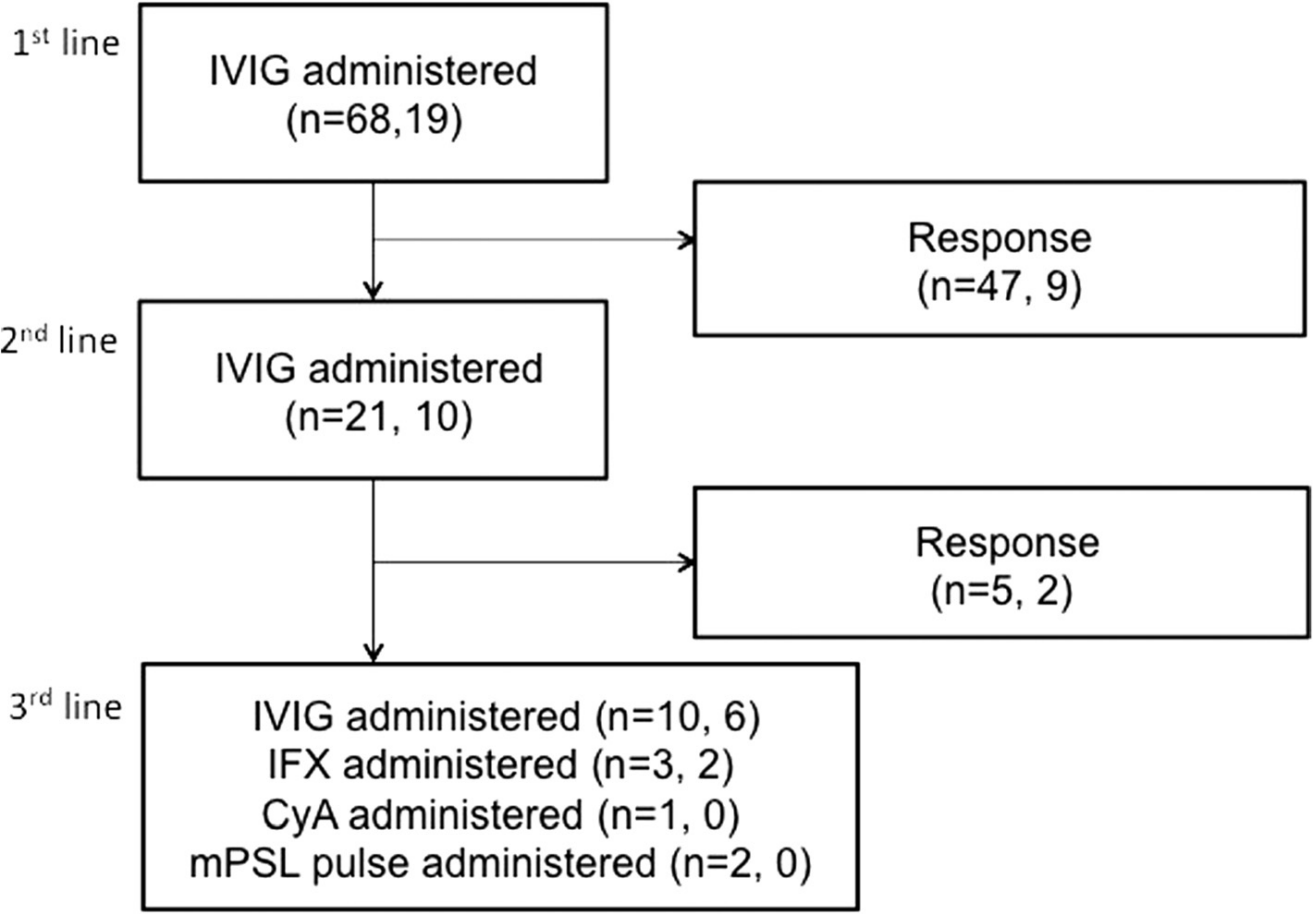
Flow chart showing the distribution of study subjects and medications used for KD in the conventional group. The numbers in parentheses denote the overall number of patients in the conventional group, and the number of patients in the conventional group whose risk scores were ≥5 points, respectively. IVIG: intravenous immunoglobulin, IFX: infliximab, CyA: cyclosporin, mPSL: methylprednisolone

**Fig. 3:**
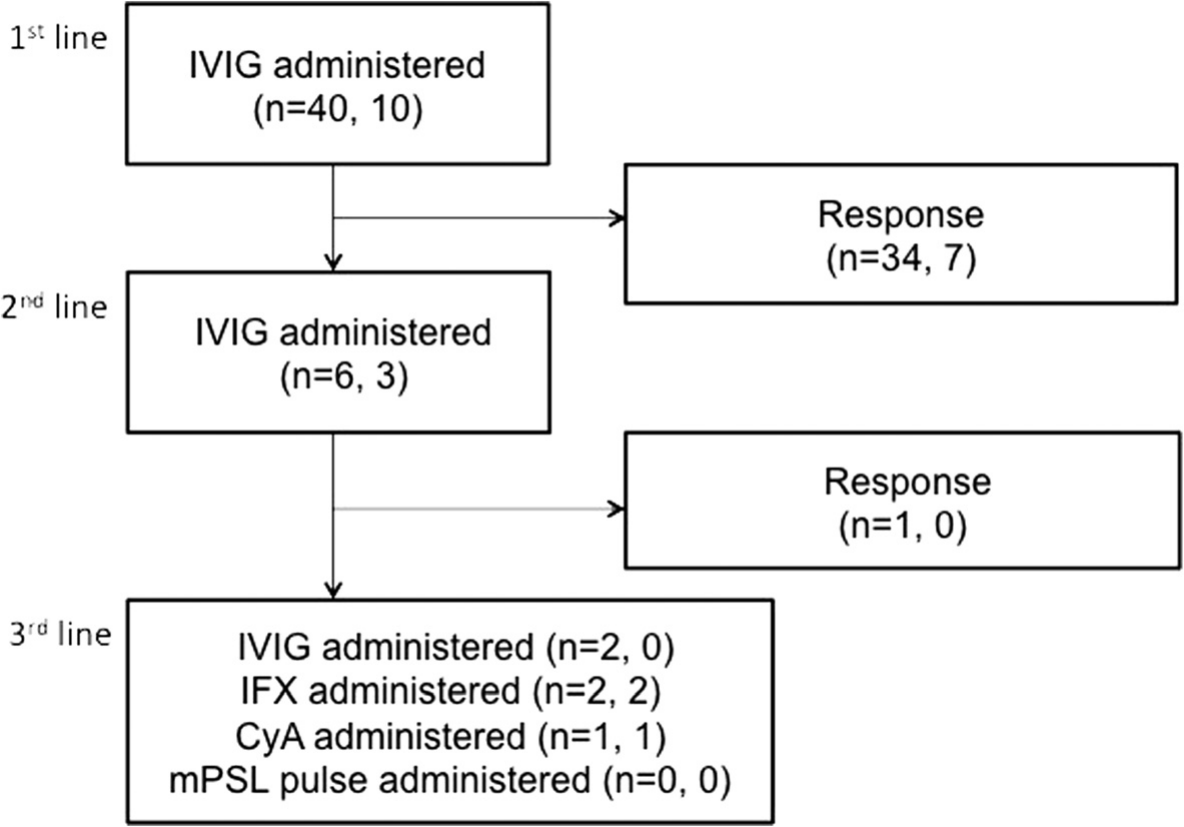
Flow chart showing the distribution of study subjects and medications used for KD in the RAISE group. The numbers in parentheses denote the overall number of patients in the RAISE group, and the number in the RAISE group who received PSL because of risk scores ≥5 points, respectively. IVIG: intravenous immunoglobulin, IFX: infliximab, CyA: cyclosporin, mPSL: methylprednisolone

Figures 2 and 3 show the medications and the number of patients overall, whose risk scores were ≥5 points, in the conventional group and the RAISE group, respectively. Forty of 108 patients were treated according to the RAISE study protocol [15] (RAISE group). Ten patients in the RAISE group had positive risk scores and received IVIG + PSL therapy (Fig. 3). As shown in Table 3, there were no significant differences in clinical characteristics between patients in the RAISE and conventional groups. Two of 40 patients (5 %) in the RAISE group and one of 68 (1.47 %) in the conventional group had CS (*p* = 0.55). Further, two of 10 patients (20 %) with positive risk scores in the RAISE group (one YPT positive and one YPT negative) and one of 19 patients (5.26 %) with positive risk scores in the conventional group (YPT positive) had CS (*p* = 0.27).

**Table 3:** Characteristics of patients according to study group

	RAISE group (*n* = 40)	Conventional group (*n* = 68)	*P* value
Sex (male/female)	23/17	44/24	0.54
Age (months)	29 (16.25–47.75)	23 (13–51.75)	0.44
Duration of fever (days)	5.5 (5–6.75)	6 (5–8)	0.21
Frequency of administration of IVIG	1 (1–1)	1 (1–2)	0.11
Acute-phase coronary artery dilation; n (%)	3 (7.5 %)	3 (4.41 %)	0.67
CS; n (%)	2 (5 %)	1 (1.47 %)	0.55
Abdominal symptoms; n (%)	18 (45 %)	23 (33.82 %)	0.31
White blood cell count (/*μ* L)	13,250 (11,700–17,200)	12,700 (10,200–15 100)	0.057
Absolute neutrophil count (/*μ* L)	8547 (7195–11,520)	7482 (5356–10,086)	0.057
Serum albumin concentration (g/dL)	3.7 (3.4–3.9)	3.7 (3.3–4)	0.53
Serum total bilirubin concentration (mg/dL)	0.6 (0.4–0.775)	0.6 (0.4–0.8)	1
Serum aspartate aminotransferase (U/L)	37 (28–80.75)	29 (22–71)	0.079
Serum sodium concentration (mmol/L)	136 (134–138)	136 (134–137)	0.12
Serum CRP concentration (mg/dL)	7.6 (3.0925–12.545)	6.36 (2.52–11.08)	0.41
Serum procalcitonin concentration (ng/mL)	0.8 (0.245–2.5125)	0.405 (0.1625–6.317)	0.074
N-terminal pro-brain natriuretic peptide (pg/mL)	336 (146–811.5)	510 (130–983.5)	0.55
Soluble interleukin-2 receptor (U/mL)	1585 (1087.5–2090)	1660 (1070–2360)	0.59
Urinary *β* 2-microglobulin/creatinine ratio	19.36 (5.92–151.9)	21.57 (5.97–130.74)	0.98
YPT positive group	6 (15 %)	5 (7.35 %)	0.32
High risk patients; n (%)	10 (25 %)	19 (27.9 %)	0.82
High risk YPT positive; n (%)	2 (5 %)	1 (1.47 %)	0.55
High risk patients with CS; n (%)	2 (20 %)	1 (5.26 %)	0.27
High risk, YPT positive, CS; n (%)	1 (50 %)	1 (100 %)	1

Values expressed as count (%) for categorical variables and median (IQR) for continuous variables

These findings indicate that the RAISE study treatment protocol (IVIG + PSL for high-risk patients with no response to IVIG and IVIG for low-risk patients) did not decrease the frequency of CS in our cohort. Additionally, YPT infection did not affect risk scores of no response to IVIG in either RAISE or the conventional period.

## Discussion

This is the first study to comprehensively investigate KD patients for evidence of YPT infection by assessing both anti-YPT antibodies for serotypes that can infect humans and YPM antibodies. We also analyzed the effect of the RAISE regimen to prevent CS development in KD.

This study yielded four important clinical observations. First, 11/108 KD patients (10 %) had positive titers of anti-YPT and/or anti-YPM antibodies. Second, the incidence of CS was significantly higher in the serologically positive (two patients, 18 %) than in the negative group (one patient, 1 %) (*p* = 0.027). Third, there were no clinical differences between the serologically positive and negative groups, except for the incidence of abdominal symptoms and CS. Fourth, in our cohort, the use of the RAISE study protocol, comprising IVIG + PSL therapy, did not decrease the frequency of CS in high-risk patients, neither did YPT infection influence sensitivity to treatment.

In 1983, Sato et al. reported that 2/12 patients with YPT fulfilled the criteria for KD [20]. Since then, several other groups have confirmed an association between YPT infection and KD. Baba et al. reported that, from 1981 to 1989, 29/329 of their YPT patients fulfilled the criteria for KD [5]. In 2006, Tahara et al. reported that, from 1985 to 2004, 42/452 of their KD patients had bacteriological culture and/or serological evidence of YPT infection [7]. Although the percentage of KD patients with YPT infection in our study is similar to that in previous reports, the ratio may differ in other regions because geographical heterogeneity of YPT strains has been reported [21, 22].

We found increases in more than two types of antibodies in three patients (Patients 7, 9 and 11; Table 1). These findings may reflect-specific responses; however, two of these three patients were also positive for anti-YPM antibodies (Patients 9 and 11), which makes non-specific reactions unlikely. A search of published reports revealed no reports of cross-reacting antigens between different strains of YPT or of co-infection with more than two types of YPT in humans. Fukushima et al. described a patient infected with O serotype-untypeable strains with positive titers for two serological types of YPT [11]. Additionally, discrepancies between classical serotypes and O-genotypes have been reported [8]. Thus, there may be some new or untypeable strains of YPT.

Three of 11 patients in the serologically positive group (27 %) were positive for anti-YPM antibodies and two of three for both anti-YPT and anti-YPM antibodies. In 1993, Abe et al. demonstrated that YPT produce a superantigen, YPM [23, 24]. In 1995, Yoshino et al. reported that 95 % of YPT strains in Japan harbor the *ypm* gene [21]. In 1997, Abe et al. reported detection of anti-YPM antibodies in 61 % of acute phase patients with YPT infection. Patients with systemic symptoms such as lymphadenopathy, transient renal dysfunction, and arthritis had significantly higher titers of anti-YPM than patients with gastrointestinal tract symptoms alone [14]. We speculate that checking for anti-YPM antibody would increase the rate of detection of YPT in KD patients. Further studies are required to ascertain whether this is true.

We found that the incidence of CS was significantly higher in the serologically positive group (two patients, 18 %) than in the negative group (one patient, 1 %) (*p* = 0.027). In 1997, Konishi et al. reported a patient with Kawasaki disease, coronary artery aneurysms, and documented YPT type 4b infection confirmed by both microbiological and serological findings [4]. In 2006, Tahara et al. reported that 22/42 KD patients with YPT infection had coronary artery lesions, this incidence being significantly higher than in the YPT-negative group [7]. In 2014, Kusuda et al. reported that serum KD-specific molecules were mostly derived from biofilms and possessed molecular structures common to microbe-associated molecular patterns from *Bacillus cereus, Bacillus subtilis,* YPT *and Staphylococcus aureus*. They also reported that extracts from *Bacillus cereus, Bacillus subtilis,* YPT *and Staphylococcus aureus* induced proinflammatory cytokines by human coronary artery endothelial cells [25]. This pathogenesis can contribute to the higher incidence of CS in cases of KD associated with YPT.

As shown in recent published studies, new medications such as infliximab, cyclosporin, and methylprednisolone have been developed [18, 26–29]. These have reduced the incidence of CS in KD patients; however, there is still a 2.8 % incidence of CS in Japan ([http://www.jichi.ac.jp/dph/kawasakibyou/20130909/mcls22report.pdf] (in Japanese)). Our findings suggest that YPT infection is a major risk factor for CS in KD patients even after the adoption of these newly developed treatment strategies.

In this study, the only clinical distinction between KD patients with and without YPT infection was in the incidence of abdominal symptoms and CS. Of note, YPT is usually very sensitive to most antibiotics [6, 30]. Antibiotics for Group-A streptococcal infection reduce the incidence of acute rheumatic fever [31]. In the future, we should aim at conducting research to clarify if antibiotic treatment for YPT infection could prevent the development of KD or reduce the incidence of CS after rapid diagnostic techniques for YPT are developed, such as the loop-mediated isothermal amplification method [32].

In our cohort, treatment with IVIG + PSL, as described in the RAISE study, did not decrease the frequency of CS in high-risk patients. In 2012, Kobayashi et al. reported the findings of a randomized, open-label, blinded-endpoints trial in which 248 high-risk patients participated (the RAISE study). They found a significantly lower incidence of CS in patients who received IVIG + PSL therapy than in those who received conventional IVIG therapy [15]. After the publication of this study [15], the use of steroids has been spreading for the standard treatment of KD and the use of steroids are recommended for the cases who did not respond to initial treatment with IVIG as written in KD treatment guideline in Japan [18]. The recent national survey in Japan revealed that among patients not responding to IVIG therapy, 29.0 % of them were treated with steroids [19]. However, it is not a routine practice in other countries because the evidence for the use of steroids is generally lacking in non-Japanese populations [33, 34]. Although RAISE protocol is widely prevalent in Japan, further larger randomised controlled trials are required to evaluate the use of corticosteroid therapy for both adjunctive primary treatment in high-risk cases and for additional ‘rescue’ therapy in IVIG non-responsive cases [33].

In our cohort, there were fewer high-risk patients (*n* = 29) and only ten of them received IVIG + PSL. However, two of the ten high-risk patients who received IVIG + PSL therapy had CS, indicating that even this treatment strategy did not completely prevent CS in all high-risk KD patients.

One limitation of our study is that no YPT was cultured from patients’ blood and/or stool samples after they had been kept in cold enrichment cultures at 27 °C. This may be attributable to antibiotic administration before obtaining the samples, which occurred in about 60 % of our KD patients. Thus, our study lacks definitive evidence of YPT infection in the patients with KD who were serologically positive. Another limitation is that, although this was a single-center study, our therapeutic strategy changed during its course because of the publication of the RAISE study, which showed clear evidence of the efficacy of steroids for KD. Further, this study included relatively few YPT positive patients overall and relatively few high-risk patients.

## Conclusions

We documented that 10 % of our KD patients had positive anti-YPT and/or anti-YPM antibody titers and 18 % of serologically positive patients had CS, meaning that YPT infection is associated with a very high-risk of CS in patients with KD. We failed to detect any other clinical differences between serologically positive and negative patients, aside from gastrointestinal symptoms. We also reported that treatment with IVIG + PSL as reported by the RAISE study did not decrease the frequency of CS in this study. However, our sample size was overly small to draw such conclusions. Further investigation in a larger cohort is necessary to confirm our findings.

Though many studies on KD treatment have been performed, few of them have focused on the cause of this condition. Further studies are needed to clarify the effect of YPT infection on KD and identify the cause of KD and its optimal treatment. Furthermore, detection of YPT infection and specific treatment in the early phase could reduce the risk of CS.

## Abbreviations

KD: Kawasaki disease
YPT: *Yersinia pseudotuberculosis*
YPM: *Y. pseudotuberculosis*-derived mitogen
CS: Cardiac sequelae
RAISE: Randomized controlled trial to Assess Immunoglobulin plus Steroid Efficacy for Kawasaki disease
VIG: IntraVenous ImmunoGlobulin
CRP: C-reactive protein
PSL: Prednisolone
